# TLX—Its Emerging Role for Neurogenesis in Health and Disease

**DOI:** 10.1007/s12035-015-9608-1

**Published:** 2016-01-06

**Authors:** Praveen K. Sobhan, Keiko Funa

**Affiliations:** 1 Present address: Sree Chitra Tirunal Institute for Medical Sciences and Technology, Biomedical Technology Wing, Thiruvananthapuram, Kerala 695 012 India; 2 Sahlgrenska Cancer Center, Institute of Biomedicine, Sahlgrenska Academy at the University of Gothenburg, SE 40530 Gothenburg, Sweden

**Keywords:** TLX, NR2E1, Review, Neural stem cells, Nervous system tumours, Glioma, Neuroblastoma, Neuropsychiatric disorders

## Abstract

The orphan nuclear receptor TLX, also called NR2E1, is a factor important in the regulation of neural stem cell (NSC) self-renewal, neurogenesis, and maintenance. As a transcription factor, TLX is vital for the expression of genes implicated in neurogenesis, such as DNA replication, cell cycle, adhesion and migration. It acts by way of repressing or activating target genes, as well as controlling protein-protein interactions. Growing evidence suggests that dysregulated TLX acts in the initiation and progression of human disorders of the nervous system. This review describes recent knowledge about TLX expression, structure, targets, and biological functions, relevant to maintaining adult neural stem cells related to both neuropsychiatric conditions and certain nervous system tumours.

## Introduction

*Drosophila tailless* (Tll) gene and its homolog Tlx in vertebrates belong to the NR2E subclass of orphan nuclear receptors. Tll was discovered more than two decades ago as a molecule able to specify cell fate during the embryogenesis of *Drosophila*. Recent studies on Tll and Tlx in both *Drosophila* and mouse have revealed critical roles in the developing nervous and visual systems, as well as in maintaining neural stem cells (NSC). Tll was identified as a gene needed for terminal structures in *Drosophila* embryonic development [1–3]. The Tll mutant not only lacks the posterior to the eighth abdominal segment structures (tail), but also those of the head and the brain [2, 4–6]. Similar to its phenotype, the Tll transcript expresses as symmetrical caps at both poles of the embryo [7].

In mammals, four distinct areas of the postnatal brain harbour cells with stem cell properties: (i) the subventricular zone (SVZ) [8], (ii) the subgranular zone (SGZ) [9], (iii) the subcallosal zone (SCZ) [9], and (iv) the cerebellum [10]. Neural stem cells keep the capacity to proliferate and self-renew, as well as the ability to differentiate into neuronal and glial lineages [11–14]. The NR2E1 derives from the evolutionary preserved nuclear receptor superfamily member of transcription factors (with at least 89–97 % homology). It is found in both vertebrates and invertebrates, as mentioned above [3, 15–18].

Tlx is a key regulator of NSC maintenance and self-renewal in the adult brain [19]. Expression of Tlx is specific to the neurogenic regions of the developing forebrain in several species, including the frog [16], zebra fish [17], and mouse [3]. In early neural development, NSCs increase in the neural tube over a limited number of cell cycles, expanding the size of the NSC pool by symmetric division [70]. When cells are about to determine their fate, a subset of NSCs becomes neuroprogenitors producing specialised cell types i.e. neurones, oligodendrocytes, and astrocytes. Neuroprogenitor cells are thought to have a decreased potential for self-renewal and pluripotency.

In the developing embryo and adult mouse, the protein TLX is localised to the neurogenic regions of the telencephalon, diencephalon, nasal placode, and retina [3, 18]. In the adult brain, TLX is expressed in the neural stem cells of the two neurogenetic zones—strong expression in the subventricular zone (SVZ) of the lateral ventricle and in the subgranular zone (SGZ) of the hippocampal dentate gyrus [19]. TLX has been found in proliferating neuroprogenitors of the adult SVZ, although most Tlx-expressing cells in the SVZ are quiescent [20, 21]. Tlx regulates transcriptionally the expression of multiple genes by repressing or activating target genes. Dysregulation of Tlx appears to affect the initiation and progression of human neurological disorders [22, 23], including various nervous system tumours [24–27], making TLX an interesting therapeutic target.

### Structure and Regulation of TLX

Tlx contains two structural subunits—the highly conserved DNA-binding domain (DBD), and the less conserved ligand-binding domain (LBD) [18, 28]. The TLL and TLX proteins are identical at the levels of 81 and 41 % in the DNA-binding and the ligand-binding domains, respectively [3, 18]. TLX and TLL were validated as functional homologs [18]. TLX has been recognised to act as a transcriptional repressor [29]. In the LBD region, TLX interacts with its cofactors, such as atrophin [30–32], BCL11A [33], LSD1 [34–36], histone deacetylases (HDACs) [30–32, 34, 35], and the von Hippel-Lindau suppressor protein (VHL) [25].

Analysis of the X-ray structure of human TLX-LBD [106] unveiled that TLX-LBD does not have a canonical NR structure. The LBD of the NR superfamily is composed of 12 α-helices (H) and a β-sheet forming an anti-parallel “α-helical sandwich” [107]. The human TLX-LBD lacks two LBD helices H1 and H2 that could potentially form an open LB pocket, and it folds into an auto-repressed ligand-free confirmation [105]. By using homology models of TLX-LBD, Benod and her group suggested that TLX could keep a large LB pocket, which would enable adaptation to ligands [105]. In screening for small molecules that directly bind TLX, three synthetic ligands with sufficient affinity and specificity were detected for TLX, but not for other NR2 subgroup members [105, 108]. However, the possibility remains that TLX functions independently of the ligand, regardless of whether endogenous ligands are discovered, since the structure of TLX LB pocket is similar to Nurr1 (NR4A2) which functions without ligands [108].

The functions of Tll and Tlx are affected by cofactors [14], such as transcriptional repression, being partly mediated by associations with atrophin family proteins [14, 30–32]. Yokoyama et al. [36] demonstrated that TLX binds the promoter of Pten, which is also bound by LSD1. Since Tlx-silencing hampers NSC renewal caused by LSD1 inhibitors, the LSD1-regulated self-renewal of NSCs might depend on Tlx [34]. Recently, Notch1/RBPJ were found to directly regulate transcription factors that are critical for NSC self-renewal, including Tlx, Sox2, Pax6, and Id4 [112]. The microRNA (miR) let-7d also modulates Tlx expression and activity through a conserved binding site on the Tlx mRNA transcripts [37]. Thus, in embryonic mouse brains, over-expression of let-7d inhibits NSC proliferation, promotes neuronal differentiation, and induces neuronal migration, being similar to Tlx knockdown models. Interleukin-1 beta (IL-1β) has been shown to be a negative regulator of embryonic and adult hippocampal neurogenesis, and the expression of Tlx is repressed in neuroprogenitors [6]. Conversely, IL-1β represses Tlx in differentiating newborn and mature neurones and astrocytes [38, 39]. Recently, it was demonstrated that this repression of Tlx and neuroprogenitor proliferation is mediated through the IL-1 receptor type I [40]. Another transcription factor regulating adult NSC proliferation is the sex-determining region-box 2 (SOX2), which has been shown to bind the promoter region of Tlx and activate proliferation of adult mouse NSCs [41].

### Tlx Targets

Tlx regulates a broad area of cellular activities, i.e. DNA replication, mitogen-activated protein kinase (MAPK) signal, adhesion, cell cycle, and migration [24, 27, 42, 43]. Through the conserved motifs or DNA-binding assays, the TLX targets Ascl1, Pou5f1 (Oct4), miR 137, Pax2, miR 9, Pten, Cdkn1a, Wnt7a, and Sirt1 have been identified [19, 32, 44–49]. Evidence indicates Tlx to activate Ascl1 and to promote neuronal induction in adult hippocampal neuroprogenitors [45]. During hypoxic conditions, Tlx balances the NSC commitment to the neuronal lineage, maintaining neural progenitor pools through transcriptional activation of Ascl1 and Pou5f1 expression [44, 45]. Similarly, TLX induces matrix metalloproteinase (MMP)-2, which is crucial for NSC to migrate to target areas and establish a neural network [27]. TLX can also physically bind and sequester VHL in normoxia in order to create a hypoxic environment for NSCs [25]. As a result, HIF-2α will be stabilised and recruited to the promoters of angiogenic factors i.e. VEGF and erythropoietin, both being important in maintaining NSC niches [25].

TLX represses the transcription of Pax2 [49, 50]. miR137 is a TLX target and an upstream regulator of LSD1. By recruiting LSD1 to the genomic regions of miR137, TLX represses miR137 in NSCs [35]. miR9 , another direct TLX target, forms a negative-feedback loop resulting in the modulation of Tlx expression in NSCs. This event affects the status of progenitor proliferation and differentiation [51]. Tlx is also regulated by another miR, let-7b and miR378, both of them increasing NSC differentiation [52, 113]. Emerging evidence suggests that microRNAs promote factors that induce differentiation by modulating Tlx expression in neuroprogenitor cells, and by interplay with TLX, these miRs appear to be involved in neurological disorders and neural tumours, as described below.

By analysing RNAs isolated from adult brains of wild type and Tlx mutant mice with a gene profile screen, Qu et al. [46] identified Wnt7α as a downstream target of Tlx. Wnt proteins bind cell membrane-Frizzled receptors, activating Wnt signalling pathways. Furthermore, they translocate the cytoplasmic β-catenin to the nucleus, where β-catenin binds T cell factor family (TCF) transcription factors, together activating the target genes [53]. Several TLX-binding sites are contained in the promoter region of Wnt7α, suggesting that Tlx regulates neurogenesis in an autonomous manner. The Wnt pathway has been experimentally proven to control self-renewal of NSCs.

By suppressing the G0-to-G1 cell-cycle transition, Tlx controls how progenitors proliferate and differentiate by the Pten-cyclin D1 pathway [32, 54]. As a regulator of NSC proliferation, Tlx also represses Pten in both the developing retina and the adult brain [32, 47, 54–56]. Tlx also regulates the differentiation of retinal progenitors via the phospholipase C and MAPK pathways [54]. Thus, by controlling the expression of Pten, Tlx regulates proliferation of stem cell and cell cycle re-entry during retinogenesis [47, 54, 55].

Tlx is considered to regulate NSC proliferation by governing expression of the Cip/Kip family cyclin-dependent kinase inhibitors, such as Cdkn1a (p21), Cdkn1c (p57, Kip2), and several genes downstream of p53 [19, 20, 24, 32, 42, 43, 56]. In fact, p21 and p57 are frequently expressed in differentiating neuroprogenitors [57]. The NAD-dependent deacetylase Sirt1 has been shown to co-localise with TLX in neuroprogenitors [58]. In HEK293 cells, TLX enhances the expression of Sirt1 through binding to the TLX-activating element in the Sirt1 promoter. Moreover, Tlx knockdown diminishes Sirt1 protein expression in neuroprogenitors [58]. Additionally, TLX controls the timing of the postnatal genesis of astrocytes by modulating the BMP-SMAD signalling pathway [59]. TLX binds to the enhancer region of BMP4 to repress its expression in the NSCs, and BMP4 is upregulated in nestin-expressing cells from Tlx mutant mice [59].

### Biological Functions

During the development of mouse brain, the expression of Tlx is restricted to the ventricular zone [20]. A detailed inspection of this region displayed a gradient of Tlx along the dorsal-to-ventral axis in the telencephalon [3]. Even though Tlx mutant mice display no abnormality at birth, the Tlx gene is required in embryonic brains for the building of superficial cortical layers [60]. In the cortex, the timing of neurogenesis is regulated by TLX [61] and the formation of lateral telencephalic progenitor regions [62]. Mature Tlx knockout mice display reduced cerebral hemispheres [63] and retinopathies due to deficient cell proliferation and decreased neuroprogenitors [32, 49, 50, 64]. Structures developing late in these mice are diminished in size, such as the upper cortical layers, the hippocampal dentate gyrus, and the olfactory bulbs—the active neurogenetic regions [19]. Transducing Tlx into Tlx-null cells will rescue their capacity to proliferate and self-renew [19]. Adult Tlx mutants exhibit enhanced aggressiveness, decreased copulation, epilepsy, and learning disabilities [43, 63, 65]. Conversely, in Tlx transgenic mice, hippocampal neurogenesis was stimulated, resulting in enhanced learning and memory [66]. This might suggest that the status of adult neuroprogenitors in the SGZ and the reduced neurogenesis precede neuropsychiatric conditions such as cognitive deficits and mood disorders [23], as described below.

Astrocyte markers are repressed by Tlx expression in NSCs [16], i.e. GFAP and aquaporin. The suppressor gene Pten is also involved, suggesting that the transcriptional repression is essential in maintaining their undifferentiated state [19, 32]. In the SGZ of Tlx-mutant mice, there is a significant decrease of stem cell proliferation and a deficiency in spatial learning, whereas no effect is seen on contextual fear conditioning, diurnal variation, or locomotion [43]. Retinal neuroprogenitors expresses Tlx in the mouse during retinal neurogenesis [49, 50]. In Tlx-mutant mice, the normal retinal cell types are specified early, but the number of cells in each layer later progressively decreases, ending as malformation of the vascular system [50]. Studies of conditional depletion of Tlx that will save visual function suggested that blindness at least partially is related to the cognitive defect and other behavioural abnormalities observed in these mice [43, 67, 68]. Additionally, Tlx-mutant animals suffer from defects in retinal vasculature, reaffirming the role for Tlx in the assembly of fibronectin matrices secreted by proangiogenic astrocytes [64].

### TLX and Neural Stem Cells

In the developing brain, the primary role of TLX is to prevent a precocious differentiation of NSCs [20, 61] and to maintain them in an undifferentiated state [19, 42]. These undifferentiated precursor cells are the driving force behind the formation of a complete and functional CNS [69]. TLX plays an important role in leading NSCs to the neurogenic niche [42]. The microvascular vessels of the SVZ is critical for the stem cell niche in activating and maintaining NSCs through Wnt, EGF, and other renewal and survival factors produced by the endothelial cells of blood vessels [109]. Hypoxia also plays an essential role for stem cell renewal [110], partly by enhancing the expression of Tlx [25, 27], which, in turn, binds and sequesters VHL, resulting in stabilisation of HIF-α [25]. Most of NSC expresses TLX that induces Wnt7a and increases HIF-α, resulting in stabilisation of the vasculature in the stem cell niche [111]. All these molecules synergise in maintaining the stem cell niche.

Moreover, whole-genome RNA-sequencing has revealed that Tlx coordinates many signalling pathways, regulating NSC behaviour [42]. TLX-positive cells in adult SVZ are relatively quiescent stem cells, and inactivation of TLX in these cells leads to a loss of neurogenesis in the SVZ [24, 56].

In the SVZ of the adult brain, Tlx is expressed in astrocyte-like quiescent or slowly cycling stem cells, named B cells [8, 9], being necessary for the transition of radial glia into adult NSCs [56]. Tlx is expressed both in quiescent and active transit-amplifying NSCs, C cells, in the SVZ [8, 21]. Tlx-inducible mutation eventually leads to a loss of neurogenesis in this region, whereas its overexpression leads to increased neurogenesis of NSCs. The increased neurogenesis prevents age-dependent exhaustion of NSCs. This will even make small glioma-like lesions progress to aggressive gliomas, promoted by p53 inactivation [24, 56]. This finding suggests Tlx to be a critical regulator of self-renewal in the NSCs of SVZ.

In the developing mouse embryo, Tlx emerges early at embryonic day (E) 8 in the ventricular zone, where NSCs and neuroprogenitors reside [3, 20, 61]. As cortical neurones are generated between E11 and E17, the expression of Tlx within the ventricular zone will reach its maximum at E13, diminishing by E16 [71]. In Tlx-mutant mice, neuroprogenitors undergo proliferation with shorter cell cycles from E9.5 to E12.5, resulting in precocious maturation [61]. In these mice, at E14.5, NSCs are decreased in number and undergo slower cell cycles [20]. The deficiency observed during the development of Tlx-mutant mice described above caused reduced cortical depth and size of the dentate gyrus, as well as a smaller forebrain [60]. The impaired limbic system manifests as abnormal behaviours of Tlx-mutant mice [63]. Similarly, adult NSCs, however small their population, also require Tlx in order to remain in a proliferative state. In these mice, NSCs may differentiate into glial cells expressing GFAP and aquaporin—markers of astrocytes [19].

The reduced number of cortical layer neurones and the defective limbic system found in Tlx-mutant mice are, moreover, also seen in Pax6 [72] and in T-box transcription factor (Tbr2) mutants [73]. Tbr2 mutants behave as aggressively as Tlx-mutant mice. The interaction between Tlx and Pax6 [20] facilitates formation of the pallido-subpallidal boundary, as noted above [62]. This suggests that Tlx and Pax6 cooperate to control the differentiation of radial glia into late neurogenic progenitors in the SVZ [73]. Thus, it is likely that deletion or mutation of Tlx may alter Tbr2 expression, and its deficient expression may partly lead to the defects seen in Tlx mutants.

### TLX and Disorders in the Nervous System

Recently, large genome-wide studies have revealed links between neuropsychiatric diseases and gene variations. As mentioned elsewhere, loss of the Tlx (NR2E1) gene in mice leads to thinner superficial cortical layers and fewer subsets of GABAergic interneurones in the neocortex [65]. Mutant adult mice display microencephaly, involving hypoplasia in the olfactory bulbs, entorhinal cortex, amygdala, hippocampus, and part of the medial temporal lobe [65]. These animals are aggressive and exhibit decreased anxiety as well as signs of reduced memory. As expected, Tlx transgenic mice demonstrate higher hippocampal neurogenesis, resulting in better learning and memory [66]. Prior to resident-intruder challenges, the aggressive behaviour of Tlx-mutant mice was diminished by a selective 5-HT_2A/C_ receptor antagonist. This suggests that this 5-HT_2_ receptor is involved in the mechanism behind aggression [68]. As for the basis of conditioned emotional responses, inactivation of the amygdala will prevent fear conditioning to both cue and context, whereas hippocampal dysfunction prevents fear conditioning to context only [74, 75]. Thus, both amygdala and hippocampus may be affected by the loss of Tlx. Dysfunctions of the limbic system are implicated in several psychiatric disorders [65].

Mutation of the NR2E1 gene has been related to microcephaly. Studies have been carried out on coding, un-translated, and regulatory sequences, as well as on evolutionary conserved non-coding regions [22]. Alterations were observed in the non-coding regulatory region, and a number of candidate mutations have been identified in patients with severe cortical disorders [22]. Interestingly, the evolutionary constraint in the coding region of NR2E1 was strong when compared to many other genes examined for gene diversity.

The NR2E1 region on chromosome 6q21-22 has been related to bipolar disorder (BP), especially in patients with manic episodes and a distinct heredity (bipolar I). This link has also been described in schizophrenia (SZ), as well as in some neurological disorders [76–78]. In addition, a meta-analysis of BP demonstrated the strongest genome-wide linkage at 6q21-22 [79]. Profiling of miR expression in SZ and BP post-mortem brains as well as genome-wide association studies (GWAS) have indicated miRs in the aetiologies of these disorders [80]. TLX is targeted by several miRs, among which miR137 displayed the highest degrees of gene variation in a GWAS study of SZ and BP [81]. Administration of lithium and other mood stabilisers seems to downregulate the miR let-7 family in both treatment responders and non-responders [81]. The let-7 family has also been suggested to affect synaptic development [82], and its expression in the brain can be modulated by sleep deprivation [83]. miR9-3p has been reported to associate with SZ as well [84]. The miR field is continuously expanding as new non-coding RNAs are being discovered. Associations between miRs and TLX are likely to be increasingly found in neuropsychiatric disorders. Moreover, these miRs might be used as therapy targets for regulation of Tlx in both positive and negative ways, dependent on the specific target and function.

### TLX and Tumour Stem Cells

**Fig. 1:**
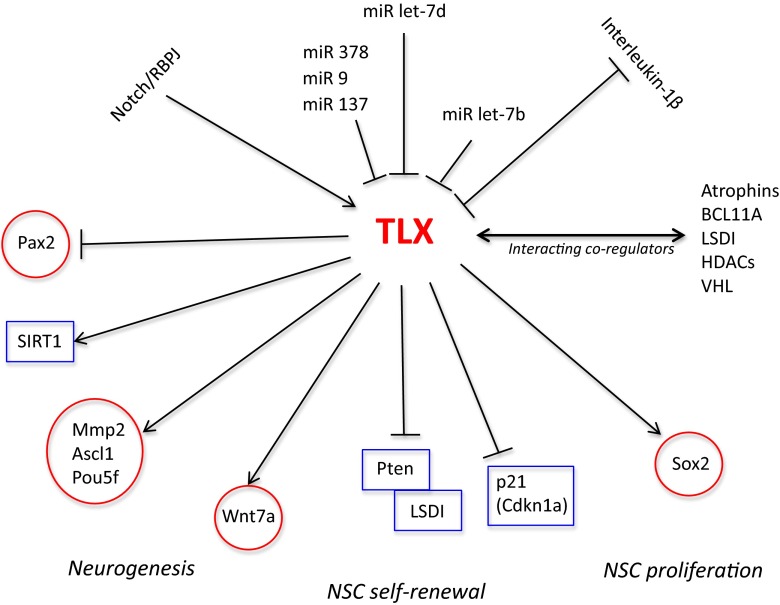
Relationships between TLX and its upstream, downstream, and interacting molecules are illustrated. Their hierarchical relations are indicated by *arrow lines* (*positive*) and *cut lines* (*negative*). Molecules in *rectangular boxes* indicate repressors, and *circular boxes* activators

Several studies indicate that brain tumour stem cells (BTSCs) may derive from NSCs. The most striking supporting evidence is that there are numerous similarities between these cell types. Accumulating evidence also indicates that different malignant tumours, including brain cancers, contain cells that maintain features of tissue-specific stem cells and are malignant [85–90]. BTSC expresses nestin, Musashi-1, Sox2, and MELK, all of which are also expressed in adult NSC [85, 89–91]. Cancer stem cells (CSCs) are capable of self-renewal and multipotent lineage differentiation, and these cells have been isolated from numerous tumours. Particularly for brain tumours, research groups have advocated that a hierarchical concept of tumour development [87, 91] is applicable in many cases. BTSCs are at the top of the hierarchy similar to NSCs, and are able to self-renew and differentiate—which might go wrong (Fig. 1) [87].

In aggressive brain tumours, the existence of BTSC was demonstrated using a nucleostemin-based murine model [92]. Further evidence has been presented describing CSCs to exist within ependymomas [91], glioblastoma multiforme [93], astrocytomas [94], and medulloblastomas [87]. What mechanisms lead to NSC transformation? For instance, ependymomas have been shown to be derived from CSC and to carry localization-specific radial glia phenotypes [91]. Additional evidence for the hypothesis that BTSCs are derived from NSCs evolves from the fact that numerous brain tumours develop in the SVZ where NSCs are located [93, 100]. Indeed, Tlx is expressed in adult NSCs [19, 21, 56, 74, 93]. The high degree of similarity between NSCs and BTSCs is a potential source of inspiration in identifying efficient targets and in designing novel treatments.

In glioblastoma, many of the signalling pathways that control stem cell development are aberrant as for the EGF receptor and the PDGF α-receptor. Other examples of stem or progenitor cell determinants being up-regulated and maybe playing a role in gliomagenesis include the transcription factors Tlx [93], Oct4 [95], Olig2 [96], and the stem cell markers, i.e. CD15, CD133, and nestin [87, 97–99], as well as markers of different neuroepithelial cells.

As for the peripheral nervous system (PNS) tumours, over-expression of TLX has been demonstrated in high-risk neuroblastoma (NB)—the childhood tumour of embryonic origin derived from the adrenosympathetic branch of neural crest cells (NCCs) [27]. When tumour-initiating cells (TICs) are enriched by tumour spheres, Tlx expression increases [27], and the side population cells are enriched (in manuscript). NCCs are highly plastic and mobile cells, being able to form both neuroepithelial and mesenchymal cells. Thus, tumours derived from NCC, such as NB and melanoma, maintain these properties, making them difficult to target.

The overexpression of Tlx alongside with common genetic lesions (e.g. mutant p53) will induce gliomas [24, 93, 101]. Tlx-GFP reporter mice overexpressing PDGFB and AKT were used to develop gliomas with differential GFP expression [26]. When compared to cells not expressing Tlx-GFP, cells doing so were largely quiescent, but they could self-renew and showed an increased sphere formation with tumour-promoting potential. Remarkably, Tlx-GFP-expressing cells were different from cells that express other putative CSC markers (i.e. Sox2 and Olig2). This may suggest that tumours may contain variable CSCs and/or stem and progenitor cells. These observations suggest that Tlx is a potential molecular target of gliomas [26, 92].

The tumour-suppressor gene Pten was identified as a TLX target during a global gene expression in a profiling study [42, 43]. Pten and p21 are likely to be the responsible genes for Tlx-induced glioma. Loss of Pten and p53 in adult NSCs leads to expansion of these cells, resulting in the formation of glioblastoma. Cell-cycle regulators and neuronal differentiation genes, such asTGFbR1 and Dlx2, were upregulated in Tlx knockout CSCs [42, 43]. These findings agree with prior reports that Tlx represses transcription in controlling CSCs. HDACs are required for this function of Tlx, which is essential for maintaining CSC self-renewal [35]. Thus, HDAC inhibitors may target CSCs to overexpress Tlx.

Since Tlx-amplified NSCs in combination with other genetic alteration contribute to tumorigenesis in the nervous system, Tlx may become a good diagnostic marker and therapeutic target for patients with malignant gliomas. In fact, Tlx is overexpressed in human glioma and neuroblastoma, suggesting that Tlx is involved in human neural tumour development. Tlx expression has furthermore been shown to correlate with poor prognosis and shorter survival of these tumours [24, 27, 56, 101]. Indeed, conditional ablation of Tlx-expressing cells will slow tumor growth and inhibit CSC self-renewal, which is associated with induction of senescence and neurogenetic differentiation [26, 102].

It is important to identify specific CSC targets, since CSCs contribute to treatment resistance [103]. A recent investigation of the full Cancer Genome Atlas dataset (that includes other prognostic factors) indicated that the prognostic importance of Tlx is related to its low expression of the glioma CpG island methylator phenotype (G-CIMP), with a single mutation of isocitrate dehydrogenase 1 (IDH1). Gliomas associated with IDH1 mutation are considered to be a genetically distinct entity predicting better survival [104]. Thus, Tlx may be a poor prognostic factor in itself. Nevertheless, its low expression in G-CIMP patients might reveal the biology in one distinct population of tumours. Future research will demonstrate the importance of CSCs, highlighting Tlx as a novel glioma CSC marker.

## Conclusion

It is urgent to examine different subtypes of malignant gliomas as for other genomic alterations with amplified TLX. Are there any mutations in the TLX gene? These questions are also relevant for neuropsychiatric disorders, which are usually considered to be triggered by combinations of multiple gene variations and epigenetics—such as methylation and changes in regulatory RNAs. Moreover, nuclear receptors interact with a variety of proteins, enabling protein stabilisation, activation, or degradation. No endogenous ligands or inhibitors of TLX have as yet been identified, but TLX is indeed a druggable target since synthetic ligands bind to the TLX-LBP [105]. Understanding the mechanisms of NSC renewal will provide insights into basic science as well as offer clinically useful replacement therapies for several disorders in the nervous system, including tumours. Mapping the TLX-controlled network that regulates these outcomes will be a major step forward in our understanding of NSC self-renewal, neurogenesis, and possibility to eradicate cancer stem cells.
